# Identification of the Genes Involved in *Riemerella anatipestifer* Biofilm Formation by Random Transposon Mutagenesis

**DOI:** 10.1371/journal.pone.0039805

**Published:** 2012-06-29

**Authors:** Qinghai Hu, Yinyu Zhu, Jing Tu, Yuncong Yin, Xiaolan Wang, Xiangan Han, Chan Ding, Beimin Zhang, Shengqing Yu

**Affiliations:** 1 Shanghai Veterinary Research Institute, Chinese Academy of Agricultural Sciences, Shanghai, People’s Republic of China; 2 China National Engineering Technology Research Centre for Poultry, Shanghai, People’s Republic of China; 3 College of Veterinary Medicine, Nanjing Agricultural University,Nanjing, People’s Republic of China; University of Wisconsin-Milwaukee, United States of America

## Abstract

*Riemerella anatipestifer* causes epizootics of infectious disease in poultry that result in serious economic losses to the duck industry. Our previous studies have shown that some strains of *R. anatipestifer* can form a biofilm, and this may explain the intriguing persistence of *R. anatipestifer* on duck farms post infection. In this study we used strain CH3, a strong producer of biofilm, to construct a library of random Tn4351 transposon mutants in order to investigate the genetic basis of biofilm formation by *R. anatipestifer* on abiotic surfaces. A total of 2,520 mutants were obtained and 39 of them showed a reduction in biofilm formation of 47%–98% using crystal violet staining. Genetic characterization of the mutants led to the identification of 33 genes. Of these, 29 genes are associated with information storage and processing, as well as basic cellular processes and metabolism; the function of the other four genes is currently unknown. In addition, a mutant strain BF19, in which biofilm formation was reduced by 98% following insertion of the Tn4351 transposon at the *dihydrodipicolinate synthase* (*dhdps*) gene, was complemented with a shuttle plasmid pCP-dhdps. The complemented mutant strain was restored to give 92.6% of the biofilm formation of the wild-type strain CH3, which indicates that the *dhdp* gene is associated with biofilm formation. It is inferred that such complementation applies also to other mutant strains. Furthermore, some biological characteristics of biofilm-defective mutants were investigated, indicating that the genes deleted in the mutant strains function in the biofilm formation of *R. anatipestifer*. Deletion of either gene will stall the biofilm formation at a specific stage thus preventing further biofilm development. In addition, the tested biofilm-defective mutants had different adherence capacity to Vero cells. This study will help us to understand the molecular mechanisms of biofilm development by *R. anatipestifer* and to study the pathogenesis of *R. anatipestif*er further.

## Introduction

*Riemerella anatipestifer* infection causes primarily a disease of domestic ducks and geese; it also occurs in turkeys, and in various other domestic and wild birds [1]. It is probably the most economically important infectious disease of farmed ducks worldwide. The disease occurs as an acute or chronic septicemia characterized by fibrinous pericarditis, perihepatitis, airsacculitis, caseous salpingitis, and meningitis [1]. Currently, at least 21 serotypes of *R. anatipestifer* have been identified [2], [3]. Once the disease has invaded duck and goose flocks, it can become endemic. Eradication is difficult, with repeated episodes of infection possible [4]. Until now, there has been little work on the molecular basis of the pathogenesis of *R. anatipestifer*, and so far no virulence factors have been identified except for OmpA [5].

The role of biofilms in the pathogenesis of some chronic human infections is now widely accepted [6]. Moreover, bacterial biofilm is a common cause of persistent infections [7], and biofilm development is an important component of bacterial survival [8]. Our previous studies have shown that *R. anatipestifer* can produce biofilm in culture. The bacteria in biofilms showed more resistance to antibiotic and detergent treatments than planktonic bacteria; therefore, biofilm may be an important reservoir of *R. anatipestifer* in packing materials or other fomites on duck farms [9].

Several studies have used the whole genome approach successfully to identify genes involved in biofilm formation, using random transposon mutagenesis in bacterial pathogens such as *Escherichia coli* O157: H7 [10], *Salmonella enteritidis* [11], *Moraxella catarrhalis* [12], *Staphylococcus aureus* [13], *Klebsiella pneumoniae* [14], [15], *Pseudomonas aeruginosa* [16] and *Cronobacter sakazakii* [17]. In this study, we report a system of random transposon mutagenesis using Tn4351 in *R. anatipestifer* for the first time; approximately 2,520 inserted mutants of strain CH3 were isolated. Subsequent characterization using crystal violet staining revealed 39 mutants defective in biofilm formation. The genes involved in biofilm formation were identified and some biological characteristics of the biofilm-defective mutants were investigated.

## Results

### Construction of a Transposon Insertion Mutant Library in *R. anatipestifer*

The transposon Tn4351 derived vector pEP4351, which has been used widely in *Bacteroides*, was used to construct an insertion mutant library in CH3 by biparental mating with BW19851 (pEP4351) and CH3. Tn4351 carries an *erm*F gene that confers erythromycin resistance on *Bacteroides* and a *tet*X gene that works only in aerobically grown *E. coli*. The *tet*X gene cannot be used as a selectable marker in *Bacteroides*, nor in *R. anatipestifer* (data not shown). On the other hand, *R. anatipestifer* CH3 has been demonstrated to be an erythromycin-sensitive strain, so we chose erythromycin resistance as a selectable marker for transconjugants. Fortunately, we succeeded in developing transposon mutagenesis in *R. anatipestifer*. A mutant library that included 2,520 transposon insertion mutants was generated in *R. anatipestifer* strain CH3. The Tn4351 transposon was integrated into *R. anatipestifer* by conjugation with *E. coli* BW19851 (pEP4351) at a frequency of 10^–6^ erythromycin resistance transconjugants per recipient cell.

### Identification of Biofilm-deficient Mutants of *R. anatipestifer*

The transposon insertion mutants were screened for altered biofilm formation using crystal violet staining. The mutants that exhibited a ≥15% reduction in OD_595_ for all three replicates of screening were characterized as biofilm-defective mutants. On the basis of this criterion, 43 out of the 2,520 transposon mutants (1.71%, 43/2520), which showed 47%–98% reductions in OD_595_, were obtained. Southern blot confirmed that 39 mutants were probed by transposon Tn4351 at one site, while the other four mutants were probed at two sites, which suggested that two copies of Tn4351 may occur in them. Further identification of the flanking sequence of transposon Tn4351 showed that there was a full pEP4351 plasmid and one insertion site in each of four mutants; therefore, these four mutants were not investigated further. Finally, a total of 39 biofilm-deficient mutants was identified (1.55%), and these mutants displayed reproducible biofilm defects.

The genes responsible for the observed defects in biofilm growth were determined by inverse PCR or genomic walking analysis of the site of the transposon insertions in these mutants. Subsequently, the sequences that flanked this site were used to conduct BLAST searches against the sequenced *R. anatipestifer* genome of strain DSM15868 (accession no. CP002346) and other nucleotides on the NCBI databases. A total of 33 mutated genes were identified. Of these, four genes were multiply mutated in different mutant strains: the *ftsA* gene was mutated in mutants BF4 and BF35, the gene encoding ribosomal large subunit pseudouridine synthase was mutated in mutants BF9, BF25, BF33 and BF39, the gene for a putative lipoprotein was mutated in mutants BF2 and BF5, and the *(p)ppGpp synthetase I* gene was mutated in mutants BF13 and BF30. Among the 33 genes mutated, 29 were located on the chromosome of DSM15868 (accession no. CP002346), the other 4 genes mutated in the mutants BF11, BF6, BF38 and BF21 were found to be on the chromosome of strain CH3 (accession numbers JN986833–JN986836), but not on that of DSM15868. These results are summarized in Table 1. Among the mutants, mutant BF19, which had a defect in the *dihydrodipicolinate synthase* (*dhdps*) gene, showed a reduction of almost 100% in the amount of biofilm formation, when compared with the amount of biofilm produced by the wild-type strain CH3, on crystal violet staining.

**Table 1:** Description of biofilm-defective *Riemerella anatipestifer* mutants.

Mutants	Locus tag (gene ID No. of DSM15868 genome)	Description of gene	Biofilm reductionrate (%)	Gene products	
				Subcellular location^a^	Function group (COGs)^b^
BF19	Riean_0023	dihydrodipicolinate synthase/N-acetylneuraminate lyase	98±2	Cytoplasmic	COG0329EM
BF12	Riean_0186	aminopeptidase N	96±4	Outer Membrane	–c
BF24	Riean_1039	cell division protein FtsQ	95±4	Cytoplasmic	COG1589M
BF34	Riean_0339	helix-turn-helix domain protein	94±5	Cytoplasmic	–
BF18	Riean_0487	AIR synthase related protein domain protein	93±6	Unknown	COG0309O
BF9, BF25, BF33,BF39	Riean_1564	ribosomal large subunit pseudouridine synthase D	91±5	Cytoplasmic	COG0564J
BF1	Riean_1987	hypothetical protein	90±7	Cytoplasmic	–
BF14	Riean_0248	phosphoribosylformylglycinamidine synthase	90±6	Cytoplasmic	COG0046F
BF28	Riean_0012	cell division protein FtsX	90±5	Cytoplasmic	COG2177D
BF11	NA^d^ (JN986833)	hypothetical protein	89±6	Unknown	–
BF20	Riean_0024	TonB-dependent receptor plug	89±4	Outer Membrane	COG1629P
B27	Riean_0263	anhydro-N-acetylmuramic acid kinase;/protein of unknown function UPF0075	89±4	Unknown	COG2377O
BF26	Riean_1092	TonB-dependent receptor plug	87±7	Outer Membrane	COG4771P
BF2, BF5	Riean_0791	putative lipoprotein	86±7	Unknown	–
BF31	Riean_0634	RimM protein	86±5	Cytoplasmic	COG0806J
BF8	Riean_1413	excinuclease ABC, C subunit	85±7	Cytoplasmic	COG0322L
BF4, BF35	Riean_1038	cell division protein FtsA	84±7	Cytoplasmic	COG0849D
BF23	Riean_0929	hypothetical protein	83±7	Outer Membrane	–
BF17	Riean_1716	1-aminocyclopropane-1-carboxylate deaminase	83±7	Cytoplasmic	COG2515E
BF16	Riean_1014	phosphodiesterase/alkaline phosphatase D	83±6	Unknown	COG3540P
BF32	Riean_1778	hypothetical protein	82±7	Cytoplasmic Membrane	–
BF22	Riean_1258	ribose-phosphate pyrophosphokinase	82±5	Cytoplasmic	COG0462FE
BF7	Riean_0335	dihydrolipoyllysine-residue (2-methylpropanoyl) transferase; pyruvate/2-oxoglutarate dehydrogenase complex	81±9	Unknown	COG0508C
BF10	Riean_0781	recA protein	81±7	Cytoplasmic	COG0468L
BF37	Riean_1893	Protein of hypothetical function DUF151	79±4	Cytoplasmic	COG1259S
BF3	Riean_0293	CzcA family heavy metal efflux protein	76±13	Cytoplasmic Membrane	COG3696P
BF29	Riean_1527	YicC-like domain-containing protein	74±9	Cytoplasmic	COG1561S
BF13, BF30	Riean_0227	guanosine polyphosphate pyrophosphohydrolases/synthetases; (p)ppGpp synthetase I, spot/rela	74±3	Cytoplasmic	COG0317TK
BF36	Riean_1769	BatA (Bacteroides aerotolerance operon); uncharacterized protein containing a von Willebrand factor type A (vWA) domain	71±5	Cytoplasmic	COG1721R
BF38	NA (JN986835)	hypothetical protein HMPREF0204_4931	64±11	Unknown	–
BF15	Riean_1174	predicted dehydrogenases and related proteins; probable oxidoreductase	61±8	Unknown	COG0673R
BF6	NA (JN986834)	autotransporter adhesin, putative outer membrane protein	56±6	Outer Membrane	–
BF21	NA (JN986836)	predicted pyrophosphatase	47±7	Cytoplasmic	–

a Subcellular locations were predicted by the PSORTb v.3.0 server. Available: http://www.psort.org/psortb/index.html. Accessed 10 June 2012.

b Functional characterization of the proteins was predicted by the software COGnitor. Available: http://www.ncbi.nlm.nih.gov/COG/old/xognitor.html. Accessed 10 June 2012. **Functional categories: (1) Information storage and processing:** (J: Translation, ribosomal structure and biogenesis; K: Transcription; L: DNA replication, recombination and repair); **(2) Cellular processes:** (D: Cell division and chromosome partitioning; O: Posttranslational modification, protein turnover, chaperones; M: Cell envelope biogenesis, outer membrane; P: Inorganic ion transport and metabolism; T: Signal transduction mechanisms); **(3) Metabolism:** (C: Energy production and conversion; E: Amino acid transport and metabolism; F: Nucleotide transport and metabolism); **(4) Poorly characterized:** (R: General function prediction only; S: Function unknown).

c –: No related COG.

d Gene not found on the genome *R. anatipestifer* DSM15868 (accession number: CP002346), but on that of strain CH3 (accession number: JN986833-JN986836).

The slide agglutination test showed that all 39 biofilm-defective mutants could agglutinate with rabbit antiserum against serotype 1 *R. anatipestifer*. In addition, the colony morphologies of 39 biofilm-defective mutants on TSB agar were similar to that of the wild-type strain CH3, suggesting that deletion of any gene of them showed no influence on the sera agglutination and colony morphology of *R. anatipestifer* CH3.

### Bioinformatics Analysis of the Proteins Encoded by the Mutated Genes

The proteins encoded by the 33 genes identified in this study (biofilm-associated proteins) were grouped into functional classes using COGnitor software, as shown in Table 1. Of these, four proteins (12.1%, 4/33) were classified in Information storage and processing related categories (J, K, L). Cellular processes and signaling related categories (D, O, M, N, P and T) included ten (30.3%, 10/33) proteins. Five proteins (15.1%, 5/33) were represented in Metabolism related categories (C, G, E, F, H, I and Q). Poorly characterized COG groups (R and S) contained four (12.1%, 4/33) proteins. “No related COGs” (the protein is not predicted to belong to any of the currently defined COGs, or the protein is not predicted to belong to a COG composed of the minimum number of clades indicated) included ten (30.3%, 10/33) proteins.

The subcellular locations of the 33 proteins were predicted using PSORTb v.3.0 software. Of these, five (15.2%, 5/33) proteins were annotated as outer membrane proteins, 18 (54.5%, 18/33) as cytoplasmic proteins, two (6.1%, 2/33) as cytoplasmic membrane proteins and eight (24.2%, 8/33) were unknown (Table 1).

### Growth Curves of the Biofilm-defective Mutants

The growth curves of the 39 biofilm-defective mutants were determined. The growth of mutants BF4 and BF35, in which the mutated genes coded for the cell division protein FtsA, was significantly slower than that of the wild-type CH3 (p<0.05), and the growth of BF13/BF30 (Tn::Riean_0227), BF10 (Tn::*recA*), BF36 (Tn::Riean_1769) and BF28 (Tn::*ftsX*), was slightly slower than that of CH3, but no significant difference was found between them (p>0.05). The growth of the other 32 mutants, including BF19 (Tn::*dhdps*), was similar to that of their parent CH3 strain.

### Biofilm Image Profiles of Selected Mutants

**Figure 1:**
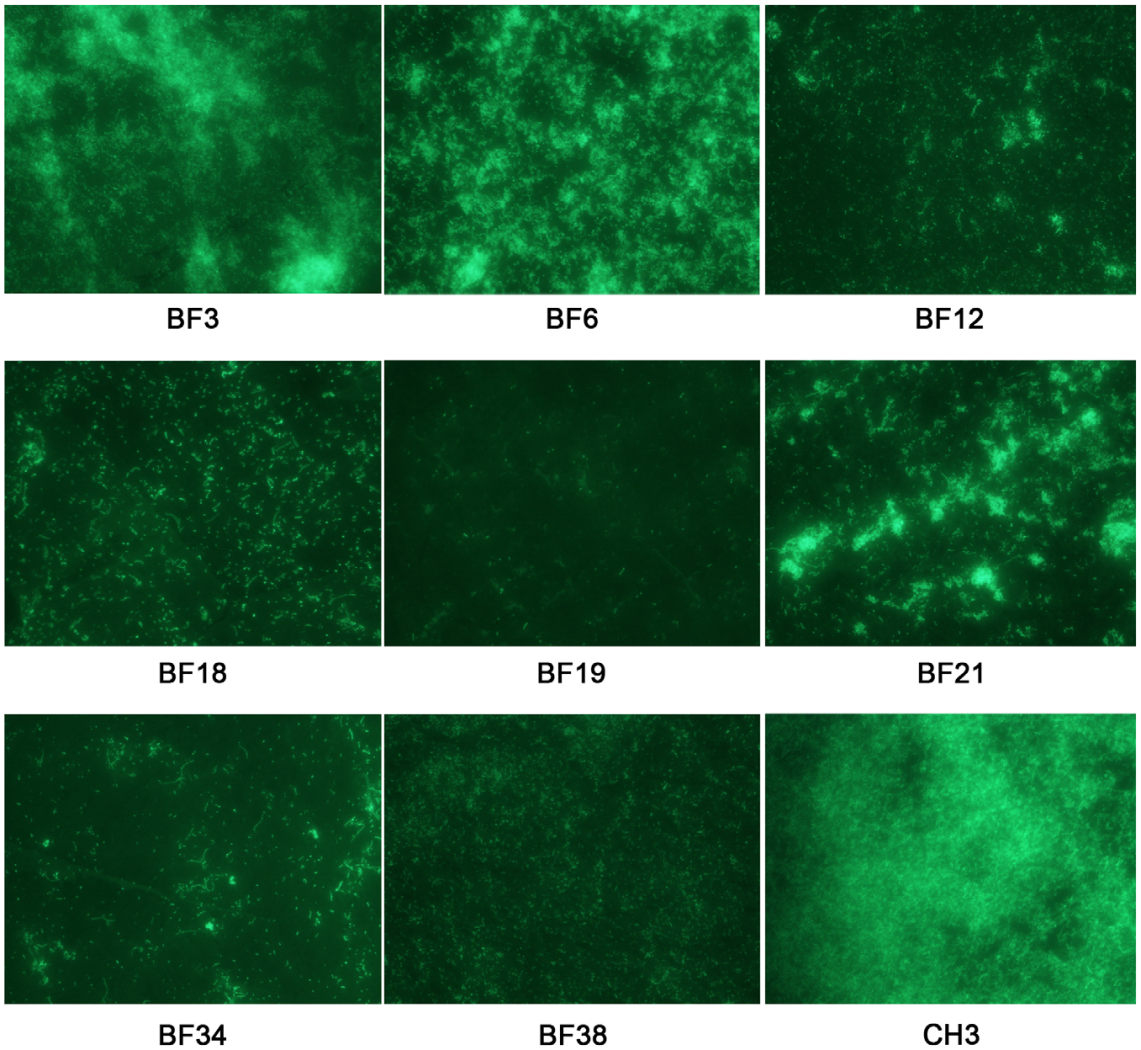
Fluorescence microscopic observations of selected biofilm-defective mutants. Biofilm images of various biofilm-defective mutants were obtained with fluorescence microscopy after staining with Live/dead BacLight Bacterial Viability staining reagent after 24 h of incubation (400×).

Eight mutants with reduced activity of biofilm formation at different levels (47±7% to 98±2%), were characterized further by comparison of their biofilms with wild-type biofilms grown on glass cover slips under fluorescence microscopic observation. The results are shown in Figure 1. After 24 h of incubation, the biofilm of wild-type CH3 was highly structured, with numerous microcolonies encased in a thick opaque extracellular matrix. For the mutant BF3, the biofilm architecture was similar to but looser than that of CH3. However, the biofilm architecture of the other seven mutants was different. For the mutants BF6, BF12 and BF21, after 24 h of incubation, some microcolonies were observed, but these were not interconnected by a homogeneous layer of bacteria like that observed with the wild-type CH3 at this stage. For the mutants BF18 and BF34, cell clusters that were formed by bacterial cells attached end-to-end, and some attached cells were observed on the glass surface. For the other two mutants, BF19 and BF38, only single attached cells were observed on the glass surface. The results indicate that the genes deleted in the mutants function on the biofilm formation of *R. anatipestifer*. Deletion of either of these genes will stall the biofilm formation at a specific stage thus preventing further biofilm development.

### Adherence Assay of Selected Biofilm-defective Mutants to Vero Cells

**Figure 2:**
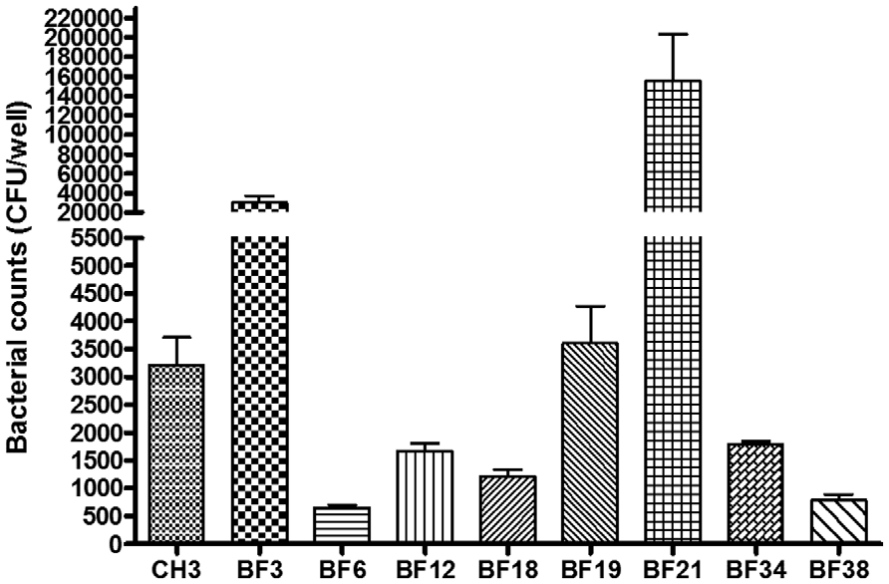
Adherence assays of selected biofilm-defective mutants to Vero cells. The data represent the number of bacteria bound to Vero cells in each well of a 24-well plate. The error bars represent means ± standard deviations from three independent experiments.

To investigate whether the mutated genes also play a role in adhesion to a biotic surface, we assessed the adherence capacity to Vero cells of the wild-type strain CH3 and eight mutants in which biofilm formation was reduced to different levels. The results are shown in Figure 2. The adherence capacity of the *dhdps* mutant (BF19) was similar to that of the wild-type CH3 (p = 0.6633, >0.05). For the mutants BF6, BF12, BF18, BF34 and BF38, adhesion was reduced significantly in comparison with that of wild-type CH3 (p<0.05). For the mutants BF3 and BF21, the numbers of bacteria recovered from the Vero cells were significantly increased compared with those of the wild-type CH3 (p<0.05).

### Determination of the Median Lethal Dose (LD_50_) of Mutants BF19, BF12, BF24 and BF34

Cherry Valley ducklings, 10 days old, were infected with four mutants and wild-type CH3 cells, respectively, to determine the effect of the mutations on the virulence of the bacterium. Ten days after infection, the calculated LD_50_ of the mutants BF12 (Tn::Riean_0186), BF19 (Tn::*dhdps*), BF24 (Tn::*ftsQ*), BF34(Tn::Riean_0339) and the wild-type strain was 1.66×10^9^CFU, 4.71×10^8^CFU, 2.73×10^8^CFU, 6.88×10^8^CFU and 2.04×10^8^CFU, respectively. The approximately 10-fold difference in the LD_50_ between BF12 and the wild-type CH3 indicated that disruption of Riean_0186 (aminopeptidase N) resulted in attenuation of the virulence of *R. anatipestifer*. The pathogenicity of BF24 (Tn::*ftsQ*) is similar to that of wild-type CH3, and the LD_50_ of the other two mutants, BF19 and BF34, was increased by two- to three-fold with respect to that of CH3.

### Complementation of Mutant BF19 (dhdps::Tn)

**Figure 3:**
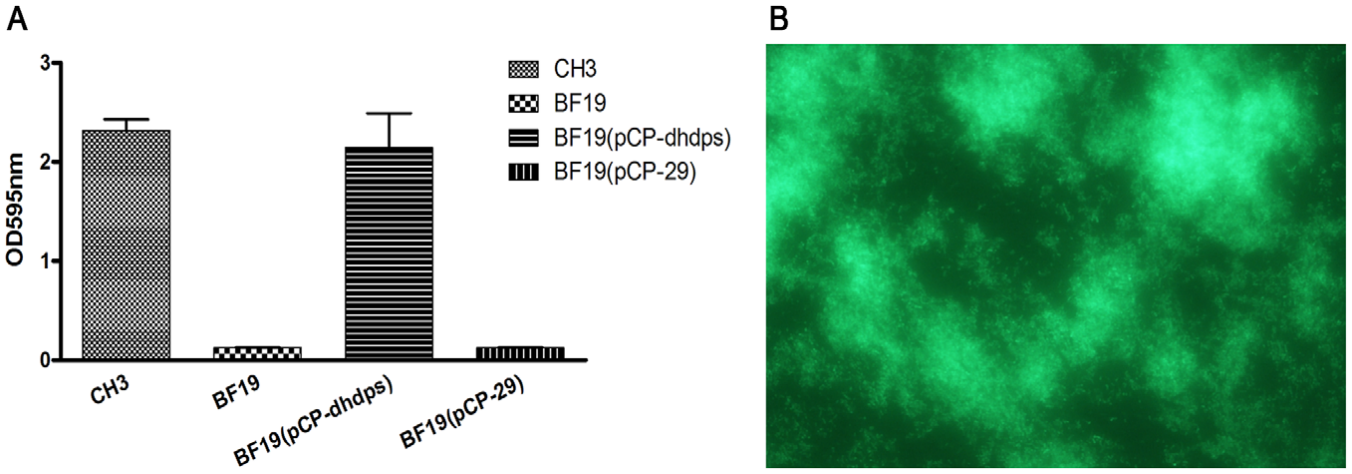
Mutant BF19 restored by the pCP29-dhdps shuttle plasmid recovered the ability to form biofilm. (A) Biofilm formation by wild-type CH3, and mutants BF19, BF19 (pCP-dhdps) and BF19 (pCP29), was measured using crystal violet staining in a 96-well microtiter plate. (B) The biofilm of complemented BF19 was observed with fluorescence microscopy after staining with Live/dead BacLight Bacterial Viability staining reagent after 24 h of incubation (400×).

For complementation of the mutant BF19 (dhdps::Tn), a plasmid, pCP-dhdps, which carries a *dhdps* gene under the control of the *R. anatipestifer ompA* promoter, was constructed and transferred from *E. coli* S17-1 into the mutant BF19 (dhdps::Tn) by conjugation. The measurement of biofilm formation by strains CH3, BF19, BF19 (pCP29) and BF19 (pCP-dhdps) using crystal violet staining showed that BF19 (pCP-dhdps) was able to produce 92.6% of the biofilm of the wild-type strain CH3 (Figure 3A). Examination of the biofilm under fluorescence microscopy also showed that biofilm formation was recovered in the complemented BF19 (Figure 3B), which indicates that the *dhdps* gene is involved in biofilm formation by *R. anatipestifer*.

## Discussion

Biofilm formation and development by bacteria has been suggested to be an important stage in the pathogenesis of numerous bacterial infections [7]. The observation that different bacterial species develop biofilms through similar stages suggests that biofilm formation is a genetically regulated process. Different proteins may be involved in the different steps of biofilm formation, and some proteins have roles in more than one step [18]. In our previous study, we found that biofilm development by *R. anatipestifer* strain CH3 involved a series of sequential steps when observed in experiments performed in vitro [9]. The 33 genes identified in this study may participate in the above steps of biofilm development by *R. anatipestifer* CH3.

The initial attachment is the first step of biofilm formation. In some Gram-negative bacteria, such as *Escherichia coli* and *Pseudomonas fluorescens*, ﬂagella and pili were found to be required to initiate the early attachment processes [19], [20]. However, *R. anatipestifer* bacterium has no ﬂagella or pili. Outer membrane proteins of *R. anatipestifer*, which have a direct role in cell–substrate or cell–cell adherence may be related to this step. In this study, five genes encoding the outer membrane proteins of *R. anatipestifer*, were identified to be involved in biofilm formation, thus these proteins may have such roles in biofilm development. Of them, the gene mutated in BF16, whose product is an autotransporter adhesion molecule, was found to be on the chromosome of strain CH3 but not on that of DSM15868, a non-biofilm producer strain (data not shown). Other four genes encoding outer membrane proteins were located also on the chromosome of DSM15868. We speculate that the product of these genes may be involved in the initial attachment step.

As some genetic factors involved in the attachment to abiotic surfaces also play a role in adhesion to both plant and animal tissues [21], [22], we analyzed the adhesion of selected mutants to Vero cells. Among them, two mutants, BF6 and BF12, in which the mutated genes coding for outer membrane proteins, and three mutants, BF18, BF34 and BF38, in which the mutated genes coding for proteins with the subcellular locations of unknown or cytoplasmic respectively, showed significantly reduced adhesion to Vero cells in comparison with that of the wild-type CH3, suggesting these genes also play a role in adhesion to Vero cells. In contrast, it was surprising that the adherence capacity of two mutants, BF3 and BF21, in which the mutated genes coding for proteins with the subcellular locations of cytoplasmic, was higher than that of CH3. Therefore, it is tempting to speculate that the mutations in these genes, which encode cytoplasmic proteins, may affect one or more adhesion factor(s) that are involved in adhesion to and aggregation on Vero cells.

Eighteen genes coding for cytoplasmic proteins and two genes coding for cytoplasmic membrane proteins were identified in this study; most of them are involved in fundamental cellular process, such as metabolism and information storage and processing. In four mutants, BF4/BF35 (*fts*A), BF28 (*fts*X) and BF24 (*fts*Q), the transposon Tn4351 was inserted into genes that are involved in cell division. Mutation of gene *fts*A in BF4/BF32 significantly reduced the growth rate and biofilm formation, compared with those of their parent strain CH3. Whether or not that the decreased growth rate of the mutants BF4 and BF35 was related to their biofilm defect remains unclear. In fact, cell division proteins (such as *fts*K) have been shown to be involved in the formation of biofilm by *Cronobacter sakazakii* [17]. Some transcription factors or protein kinases that act as regulatory proteins must function indirectly to control the properties of biofilm, and they may be informative indicators of the internal and external signals that influence biofilm development [18]. Our study also identified mutants that were defective in genes coding for transcription factors or protein kinases, such as BF13/BF30, BF22 and BF27.

The locations of another eight gene products could not be predicted by the PSORTb v.3.0 software in this study. Two of them were mutated in dehydrogenase-associated genes. Previous reports showed that both positive and negative roles in biofilm development have been found for alcohol dehydrogenases and ary-alcohol dehydrogenases [23], [24], indicating that substrate specificity is crucial to their biological function. In addition, isopropylmalate dehydrogenase is important for the survival and pathogenesis of *Burkholderia pseudomallei* [25].

Dihydrodipicolinate synthase (DHDPS) is one of the key enzymes that lead to lysine biosynthesis in some bacteria. In our study, insertion of the transposon in the *dhdps* gene (BF19) resulted in near disappearance of the capacity to form biofilm. The role of this enzyme in biofilm formation was confirmed further by the complementation experiment.

In this study, the LD_50_ of mutants BF19, BF12, BF24 and BF34 was determined. The approximately 10-fold decreased LD_50_ of BF12 indicated that disruption of Riean_0186 (aminopeptidase N) resulted in both attenuation of *R. anatipestifer* virulence and decrease of biofilm formation as well as adherence to Vero cells. In addition, the pathogenicity of BF24 (Tn::*ftsQ*) is similar to that of wild-type CH3, and the LD_50_ of BF19 and BF34 was increased about two- to three-fold. The results suggested that biofilm formation by *R. anatipestifer* was not directly related to its virulence, but some genes may be involved in both biofilm formation and the virulence of *R. anatipestifer*.

The crystal violet staining method was used to quantify the biomass of biofilm in this study. Although this is one of the standard methods used commonly for biofilm quantification, it has certain problems with reproducibility. In this study, a mutant with a reduction in OD_595_ value of ≥15% for all three replicates of screening was characterized as a biofilm-deficient mutant, as a result, 43 mutants showing 47%–98% reductions in OD_595_, were obtained. The other 8 mutants did not show ≥15% reductions in OD_595_ for all three replicates, therefore they were omitted from further experiments. The biofilm defect in the mutants was confirmed further with fluorescence microscopic observations. The biofilm images of the mutants suggested that deletion of these genes stalled biofilm formation at specific stages and thus prevented further biofilm development, and this may be of benefit for further studies on the role of the deleted genes in the development of biofilm.

In conclusion, in this study, for the first time, we developed a random Tn4351 transposon insertion library and a strategy of pCP29 shuttle plasmid-based complementation in *R. anatipestifer*. In addition, some biological characteristics of biofilm-defective mutants were investigated. Our studies have provided a significant first step towards the development of a robust set of genetic tools for the manipulation of *R. anatipestifer*. This work expands our understanding of the genetic factors that control biofilm formation and may provide potential targets for therapeutic intervention.

## Materials and Methods

### Bacterial Strains, Plasmids, and Culture Conditions

All the bacterial strains and plasmids used in this study were listed in Table 2. *Riemerella anatipestifer* CH3 is a field strain that exhibits strong biofilm formation on polystyrene surfaces [9]. The *Escherichia coli*–*Flavobacterium johnsoniae* shuttle plasmid pCP29 and *E. coli* strain BW19851, which carries the plasmid pEP4351, were provided generously by Professor Mark J. McBride at the University of Wisconsin-Milwaukee in the United States. The *R. anatipestifer* was cultured at 37°C in tryptic soybean broth (TSB, Difco, Detroit, MI, USA), and *E. coli* strains were grown routinely on Luria broth (LB, Difco, Detroit, MI, USA) agar or in LB broth at 37°C. For selective growth of bacterial strains, antibiotics were added at the following concentrations: ampicillin (100 µg/ml), chloramphenicol (5 µg/ml), erythromycin (1 µg/ml), kanamycin (50 µg/ml) and tetracycline (10 µg/ml).

**Table 2:** Strains, plasmids and primers used in this study.

Strains, plasmids or primers	Description	Source or reference
**Strains**		
CH3	*Riemerella anatipestifer* serotype 1 strain, strong biofilm-producer	[9]
*Escherichia coli* S17-1	lpir hsdR pro thi; chromosomal integrated RP4-2 Tc::Mu Km::Tn7	[32]
BW19851 (pEP4351)	Plasmid pEP4351 in BW19851, chloramphenicol resistant	[33]
CH3 (pCP29)	*Riemerella. anatipestifer* CH3 strain carrying plasmid pCP29	This study
BF19	Tn4351 insertion mutant of *Riemerella. anatipestifer* CH3, dhdps::Tn	This study
BF19 (pCP29)	Mutant BF19 carrying plasmid pCP29	This study
BF19 (pCP-dhdps)	Mutant BF19 carrying plasmid pCP29-dhdps	This study
**Plasmids**		
pEP4351	pir-requiring R6K oriV; RP4 oriT; Cm^r^Tc^r^ (Em^r^); vector used for Tn4351 mutagenesis	[34]
pCP29	ColE1 ori; (pCP1 ori); Ap^r^(Em^r^); *E. coli*-*F. johnsoniae* shuttle plasmid	[33]
pCP-egfp	pCP29 containing *dhdps* ORF under the control of the promoter of ORF1, cfxA^r^ (Ap^r^)	This study
pCP-dhdps	pCP29 containing *ompA* promoter and *dhdps* ORF, cfxA^r^ (Ap^r^)	This study
**Primers**		
340	5'-GACTTGGATACCTCACGCC-3'	[35]
341	5'-TTGGAAATTTTCTGGGAGG-3'	[35]
TN-1	5'-GGACCTACCTCATAGACAA-3'	[30]
IS4351-F	5'-TCAGAGTGAGAGAAAGGG-3'	[30]
SP1	5'-CTCCCAGAAAATTTCCAAGACTCTCA-3'	This study
SP2	5'-TAAAGTGCTGACCCGTAAAACGAAC-3'	This study
SP3	5'- GTGGTAGCTATAGCATGGAGCTTGC-3')	This study
CAT-1	5'-CACTGGATATACCACCG-3'	This study
CAT-2	5'-TGCCACTCATC GCAGTA-3'	This study
ompA promoter P1	5'-CAGGTACCATAGCTAAAATTTTGGCAGTAAC -3' (*Kpn* I site underlined)	This study
ompA promoter P2	5'-CGACTCGAGCATTCCAATTCTCTTATTATC-3' (*Xho* I site underlined)	This study
dhdps P1	5'-TACTCGAGATGAAAAATTTATCAGGTCTAGG -3' (*Xho* I site underlined)	This study
dhdps P2	5'-ATGCATGCTTAACTGAAAACAGAGTGTAGTC-3' (*Sph* I site underlined)	This study

### Construction of a Transposon Mutant Library of *R. anatipestifer*

Transposon mutagenesis was performed as described previously [26], with modifications. The *E. coli* BW19851, containing the plasmid pEP4351, was used as the donor strain and *R. anatipestifer* CH3 as the recipient. For bacterial mating, both donor and recipient cells were grown to mid-logarithmic phase, mixed at a ratio of 1∶2 (on the basis of the reading at OD_600_) and concentrated by centrifugation (5500 ×g, 10 min). The bacterial pellet was washed once with 10 mM MgSO_4_, after which the mixture was re-suspended and filtered through a Millipore membrane. The filter was placed face up on tryptic soybean agar (TSA) with 1 µg/ml erythromycin and 50 µg/ml kanamycin. Following overnight (8–10 h) incubation at 30°C, the cells were scraped off the filter, resuspended in 5 ml 10 mM MgSO_4_, and spread on TSA containing erythromycin and kanamycin to select for transconjugants. Each plate yielded 100 to 200 colonies of erythromycin-resistant mutants. A total of 2,520 independent mutant colonies were obtained.

### Screening and Identification of Biofilm-deficient Mutants

The biofilm formation of the Tn4351 insertion mutants was measured using crystal violet staining in 96-well microtiter plates, as described previously [9]. Briefly, an overnight culture of CH3 or mutant was diluted at 1∶100, and 200 µl of each cell suspension was transferred to 96-well, flat-bottomed, polystyrene plates (Corning, NY, USA) for biofilm formation and crystal violet staining respectively. The optical density at 595 nm (OD_595_) was determined using a Synergy 2 microplate reader (Biotek, VT, USA). The reduction in the rate of biofilm formation was calculated as (OD_595_ of wild type CH3– OD_595_ of a mutant)/OD_595_ of wild type CH3 × 100%. A mutant with a reduction in OD_595_ value of ≥15% [17] for all three replicates of screening was characterized as a biofilm-deficient mutant. All the mutants were screened in triplicate. To determine whether the biofilm-deficient phenotype of the mutants was due to the growth-deficient nature of the mutants, the growth curves of the biofilm-deficient mutants were measured as described previously [27].

Southern blot analysis of the Tn4351 insertions was used for the identification of the mutants. The genomic DNA from erythromycin-resistant transconjugants was isolated, digested with *Xba* I, separated by gel electrophoresis, and transferred to nylon membranes essentially as described previously [28]. The DIG DNA labeling and detection kit (Roche, Indianapolis, USA) was used to prepare probes and to perform hybridization. Two probes were used for the identification: one to detect the transposon Tn4351 and other to detect the *cat* gene, which is present on pEP4351 but not in transposon Tn4351. The *cat* gene was amplified as a 633-bp PCR product from pEP4351 using the primers CAT-1 and CAT-2. A mutant that was probed with transposon Tn4351 at one site, but could not be probed with the *cat* gene was characterized as a transposon Tn4351 mutant.

### Sequencing and Bioinformatics Analysis of Mutated Genes

The genomic DNA of the mutant strains was extracted with a TaKaRa MiniBEST Bacterial Genomic DNA Extraction Kit (TaKaRa, Dalian, China). The amplification of the DNA region at the site of Tn4351 insertion was performed using either inverse PCR or genomic walking. Inverse PCR was performed as described previously [29]. Briefly, the genomic DNA was digested with *Hind* III and then re-ligated, which resulted in the formation of circular molecules. Pairs of primers specific for Tn4351 (primer 340 and primer 341; primer TN-1 and primer IS4351-F) [30] were used to amplify the sequences adjacent to the insertion site using a TaKaRa LA PCR kit (TaKaRa, Dalian, China). Genomic walking was performed with a genomic walking kit (TaKaRa) using a variety of arbitrary primers (AP1, AP2, AP3, AP4) provided in the kit and three specific primers (SP1, SP2 and SP3), according to the manufacturer’s instructions.

Each mutated gene was PCR amplified. The PCR products were cloned into the pGEM®-T easy vector (Promega, Madison, WI, USA). DNA sequencing was performed on an Applied Biosystems DNA sequencer (ABI PRISM 3730) by Invitrogen Co, Ltd (Invitrogen, Shanghai, China). The sequences of the identified genes were searched for on the BLASTX server (http://www.ncbi.nlm.nih.gov/BLASTX/) to find homologous sequences and putative functions, and searched for using the online software PSORT v.3.0 (http://www.psort.org/) to predict the subcellular localization of the proteins. Functional characterization of the proteins was predicted using the online software COGnitor (http://www.ncbi.nlm.nih.gov/COG/old/xognitor.html), by comparison of the sequence to the Clusters of Orthologous Groups of proteins (COGs) database, which is based on COG functional categories. The sequences of genes that were not found in the genome of *R. anatipestifer* DSM15868 (accession number: CP002346), but were present in that of strain CH3, were submitted to GenBank.

### Growth Curves of the Biofilm-defective Mutants

Thirty-nine biofilm-defective mutants and wild-type strain CH3 were grown in TSB at 37°C with shaking, and the growth curves were determined as described previously [27]. The statistical significance of the data was determined by one-way ANOVA in Graphpad Prism 5 software (GraphPad Software, Inc., CA, USA). A P value of <0.05 was considered to be statistically significant.

### Fluorescence Microscopic Observations of Selected Biofilm-defective Mutants

Eight biofilm-defective mutants, BF3, BF6, BF12, BF18, BF19, BF21, BF34 and BF38, in which the biofilm formation is reduced at different levels, were selected and stained with Live/dead BacLight Bacterial Viability staining reagent at 24 h incubation. The image profiles of the biofilms were observed using fluorescence microscopy as described previously [9].

### Adherence Assay of Selected Biofilm-defective Mutants to Vero Cells

To determine whether the genes inactivated in the mutants that were associated with defects in biofilm formation had an influence on the adherence capacity of CH3 to Vero African green monkey kidney epithelial cells (ATCC CCL-81, Manassas, USA), a bacterial adherence assay was performed for eight biofilm-defective mutants, respectively, as described previously [5].

### Animal Experiment

One-day-old Cherry Valley ducks were purchased from Zhuanghang duck farm (Fengxian District, Shanghai). The ducks were housed in cages with a 12-h light/dark cycle and free access to food and water during the study. Care and maintenance of the animals were in accordance with the Institutional Animal Care and Use Committee (IACUC) guidelines set by Shanghai Veterinary Research Institute, the Chinese Academy of Agricultural Sciences (CAAS).

To determine whether the genes inactivated in the mutants that caused defects in biofilm formation had an influence on virulence, the median lethal dose (LD_50_) values of wild-type strain CH3 and four mutants, BF12, BF19, BF24 and BF34, in which the biofilm formation was reduced by 96±4%, 98±2%, 95±4% and 94±5% respectively, were measured using 10-day-old Cherry Valley ducklings as described previously [9].

### Complementation of the Mutant Strain

To determine whether the biofilm-deficient phenotype was due to an inactivated gene, a biofilm-deficient mutant strain, BF19, in which *dhdps* gene (Riean_0023) was inactivated by the insertion of Tn4351, was used for the complementation experiment. A recombinant pCP29 plasmid that contained an expression cassette consisting of the *R. anatipestifer ompA* promoter and a *dhdps* gene was constructed on the basis of the *E. coli*–*F. bacterium* shuttle plasmid pCP29. The expression of the *dhdps* gene was under the control of the *ompA* promoter. The *ompA* promoter of the CH3 strain was amplified by PCR using the primers ompA promoter P1 and ompA promoter P2, and the *dhdps* ORF was amplified using the primers dhdps P1 and dhdps P2. The PCR products of the *ompA* promoter and the *dhdps* gene were ligated into the Promega T easy vector and digested with *Kpn* I and *Xho* I. Subsequently, the two DNA fragments were ligated into pCP29 at the sites of *Kpn* I and *Sph* I to generate pCP-dhdps. The plasmid pCP-dhdps was transformed into S17-1 by a TSS method [31] to obtain S17-1 (pCP-dhdps). For complementation analysis, the plasmid pCP-dhdps was introduced into the *R. anatipestifer* CH3 mutant BF19 (dhdps::Tn) by conjugation as described previously [5], to generate BF19 (pCP-dhdps). The transconjugants were selected using TSA containing 5 µg/ml cefoxitin and identified further by PCR amplification of the *cfxA* gene on the plasmid pCP29 and the *dhdps* gene in the genome of strain CH3.

To determine whether or not the ability of the mutant BF19 (dhdps::Tn) to form biofilm would be restored when the plasmid pCP-dhdps was introduced into the mutant, biofilm formation by strains CH3, BF19, BF19 (pCP29) and BF19 (pCP-dhdps) was measured using the crystal violet staining method [6].
